# Indole-3-acetic acid improves drought tolerance of white clover via activating auxin, abscisic acid and jasmonic acid related genes and inhibiting senescence genes

**DOI:** 10.1186/s12870-020-02354-y

**Published:** 2020-04-08

**Authors:** Youzhi Zhang, Yaping Li, Muhammad Jawad Hassan, Zhou Li, Yan Peng

**Affiliations:** 1 Department of Grassland Science, College of Animal Science and Technology, Sichuan Agricultural University, Chengdu, 611130 China; 2 College of Life science, Changchun Normal University, Changchun, 130032 China

**Keywords:** Drought stress, IAA, Phytohormone, Auxin, Transcription factor, Drought responsive gene, Senescence-associated gene

## Abstract

**Background:**

Auxin may have a positive effect on plants under drought stress. White clover is widely cultivated and often prone to water shortages. In the present study, we investigated the effects of exogenous indole − 3-acetic acid (IAA) on growth and physiological changes of white clover under drought stress condition. The contents of endogenous IAA and other hormones including ABA, CTK, JA, GA, IAA, and SA were assayed. Moreover, expressions of auxin-responsive genes, drought-responsive genes and leaf senescence-associated genes were detected in response to exogenous IAA.

**Results:**

Compared to control, drought stress alone significantly diminished stem dry weigh, relative water content (RWC) and total chlorophyll content (Chl). Exogenous IAA treatment significantly increased RWC and Chl, whereas L-AOPP treatment drastically decreased stem dry weight, RWC and Chl under drought stress condition. Additionally, exogenous IAA treatment significantly increased ABA content and JA content, up-regulated expression of auxin responsive genes (*GH3.1*, *GH3.9*, *IAA8*), drought stress responsive genes (*bZIP11*, *DREB2*, *MYB14*, *MYB48*, *WRKY2*, *WRKY56*, *WRKY108715* and *RD22*), and down-regulated expressions of auxin-responding genes (*GH3.3*, *GH3.6*, *IAA27*) and leaf senescence genes (*SAG101* and *SAG102*) in the presence of PEG. Contrarily, L-AOPP treatment significantly reduced contents of ABA, GA3 and JA, down-regulated expressions of *GH3.1*, *GH3.9*, *IAA8*, *bZIP11*, *DREB2*, *MYB14*, *MYB48*, *WRKY2*, *WRKY56*, *WRKY108715*, *ERD* and *RD22*, and up-regulated *SAG101* and *SAG102*.

**Conclusions:**

Exogenous IAA improved drought tolerance of white clover possibly due to endogenous plant hormone concentration changes and modulation of genes involving in drought stress response and leaf senescence. These results provided useful information to understand mechanisms of IAA improved drought tolerance in white clover.

## Background

Water is a vital component for plants as it allows plants to survive [1]. Drought stress adversely impacts plant growth and has attracted researchers’ attention due to its effects on a variety of physiological and metabolic mechanisms in plants [2–4]. It has been estimated that up to 30% of the plants in the world are subjected to varying degrees of drought (https://www.un.org/en/events/desertification_decade/whynow.shtml). To maintain growth and development, plants adjust their morphological and physiological characteristics in response to drought, which improve plants’ ability to withstand water deprivation in arid environments [5–8]. For example, plants accumulate low-molecular-weight osmolytes to recruit potassium and other nutrient ions, and increase root elongation to enhance water uptake. Additionally, phytohormones, such as auxins (IAA), abscisic acid (ABA), cytokinin (CTK), salicylic acid (SA), gibberellin (GA) and jasmonic acid (JA), could modulate the plant tolerance to drought stress. Studies approved the alleviation of drought stress in plants after application of hormones [9–11], however, the changes of endogenous hormones were not illustrated. Among the phytohormones mentioned above, IAA regulates many processes during plant growth and development [7, 12]. Recently, accumulating evidences indicate the possible link between IAA and other hormones [13–15], indicating the cross-talks among phytohormones might play key roles during plant stress response.

To date, some drought-responsive genes have been identified, such as Early Responsive to Dehydration (*ERD*) that are rapidly activated by drought stress [16, 17] and Senescence-Associated Genes (*SAG*) that regulate chlorophyll degradation and cytoplasmic destruction [18]. In Arabidopsis, *ERD1* encodes a protein, which is induced by dehydration and does not respond to ABA [19]. *ERD10* and *ERD14* are strongly induced in Arabidopsis by dehydration and ABA, but not by 2, 4-D and GA [16, 20]. However, the effect of IAA on the expression of *ERD* is unknown. The expression of *SAG13* gene in Arabidopsis increases during senescence [21]. Therefore, the expression of *SAG* gene could be an indicator of plant senescence. Plant hormones are involved in the regulation of plant senescence. Therefore, the application of IAA possibly modulates the expression of *SAG* gene and needs to be studied.

In addition, the expression of plant transcription factor (*TFs*) genes, including basic region/leucine zipper (*bZIP*) motif, dehydration response element-binding (*DREB*), myeloblastosis (*MY*B), and *WRKYs* were also modulated after drought stress treatment [22–25]. Overexpression of *OsbZIP72* and *OsbZIP46* significantly increased the drought resistance of rice by elevating the expression of ABA response gene [26, 27]. *DREB2* has been reported to play a crucial role in enhancing the abiotic stress tolerance of plants by interacting with a cis-element present in the promoter region of various abiotic stress-responsive genes [24]. It has been reported that *PbrMYB21* could promote drought tolerance of tobacco due to the modulation of polyamine synthesis by regulating the arginine decarboxylase expression [28]. The up-regulated *OsMYB48–1* promoted drought tolerance of rice by means of regulating the expression of *OsNCED4* and *OsNCED5* (ABA biosynthesis genes), *OSRK1* and *OsPP2C68* (early signaling genes) and some late responsive genes such as OsLEA3 and RAB21 [29]. Overexpression of *ZmWRKY58,* which interacted with ZmCaM2, enhanced the drought tolerance in transgenic rice [30]. Both *TaWRKY1* and *TaWRKY33* activated several stress-related genes and promoted root growth in *Arabidopsis* under various stresses, and increased the drought resistance in Arabidopsis [25]. In previous studies, auxin also regulated gene expression of auxin responding genes [31–33] and Auxin-Response transcription Factor (*ARF*) family mediated the roles of IAA during plant growth [34]. Also, there is very little evidence available to support that auxin regulates the expresion of *ARF*as well as expressions of TFs genes.

White clover (*Trifolium repens*) is one of the essential forages. It is widely cultivated and grazed to many animals in the world due to its high yield and quality. However, it is susceptible to drought stress and shows significant changes in dehydration during drought [35]. In addition, the effects of IAA on white clover under drought stress are not fully understood. The objects of the research are to reveal: (1) whether IAA has crosstalk with other plant hormones in white clover under drought stress condition; (2) how could IAA activate auxin signal transduction pathway and modulate expressions of drought responsive genes.

## Results

### Effects of exogenous IAA on drought tolerance of white clover

**Fig. 1:**
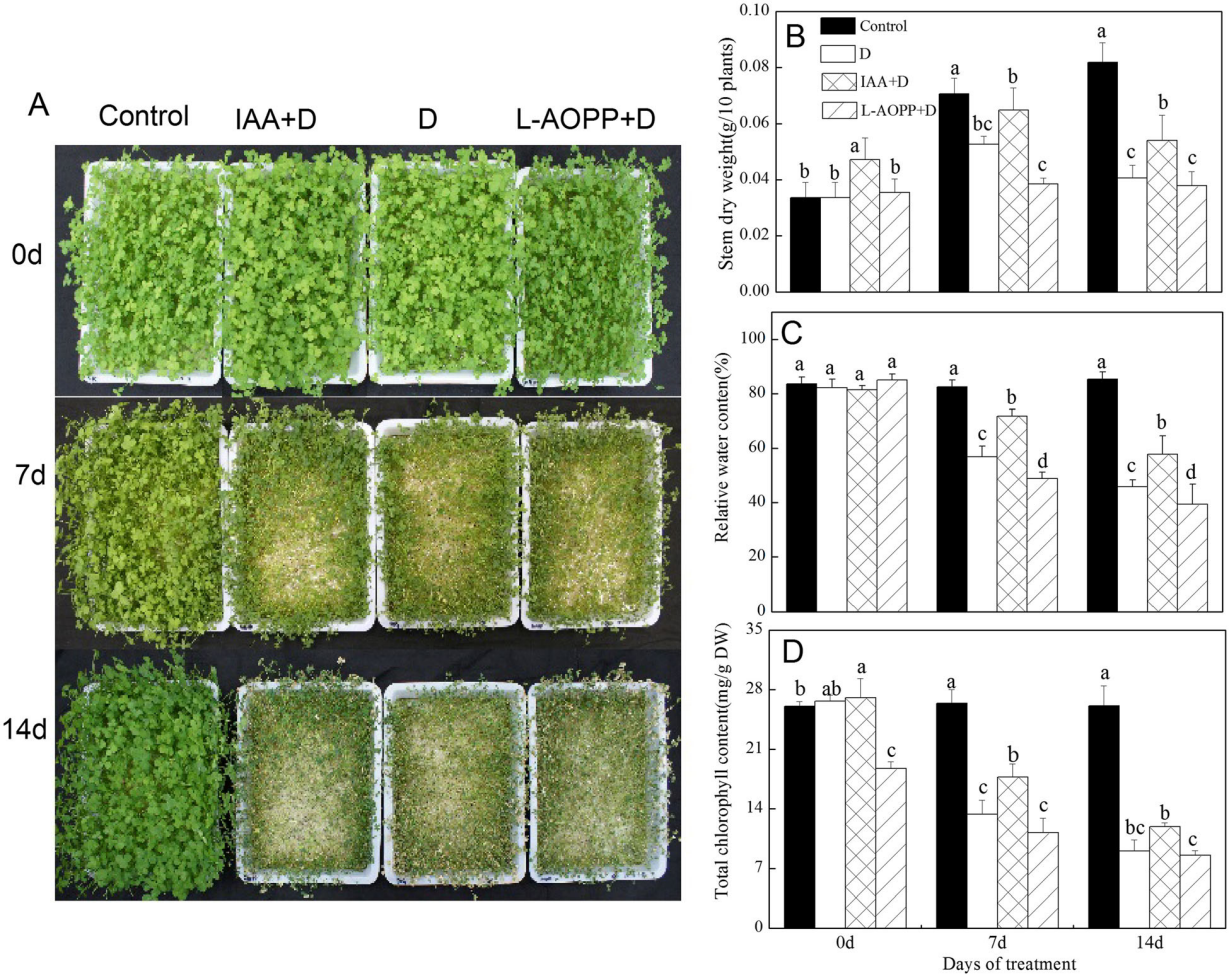
Morphological appearance (**a**), Stem dry weight (**b**), relative water content (**c**) and total chlorophyll content (**d**) of white clover in different sets. Vertical columns represent Mean + STD (*n* = 4). The same letter indicates no significant difference and the different letters indicate a significant difference (Fisher’s LSD, *P* < 0.05) in a pairwise comparison between sets at the same time

The morphological appearance of white clover was shown in Fig. 1a. On 0 d (no PEG stress), exogenous IAA significantly increased stem dry weight and Chl (Fig. 1b-d) and had no effect on RWC. L-AOPP significantly reduced Chl (Fig. 1d). On 7 d and 14 d, in all PEG sets, IAA increased stem dry weight, RWC and Chl, while L-AOPP decreased these indicators (Fig. 1b-d). Meanwhile, our results showed that RWC and Chl of white clover in control remained stable.

### Content of ABA, CTK (iPAs and ZRs), GA3, JA and SA

**Fig. 2:**
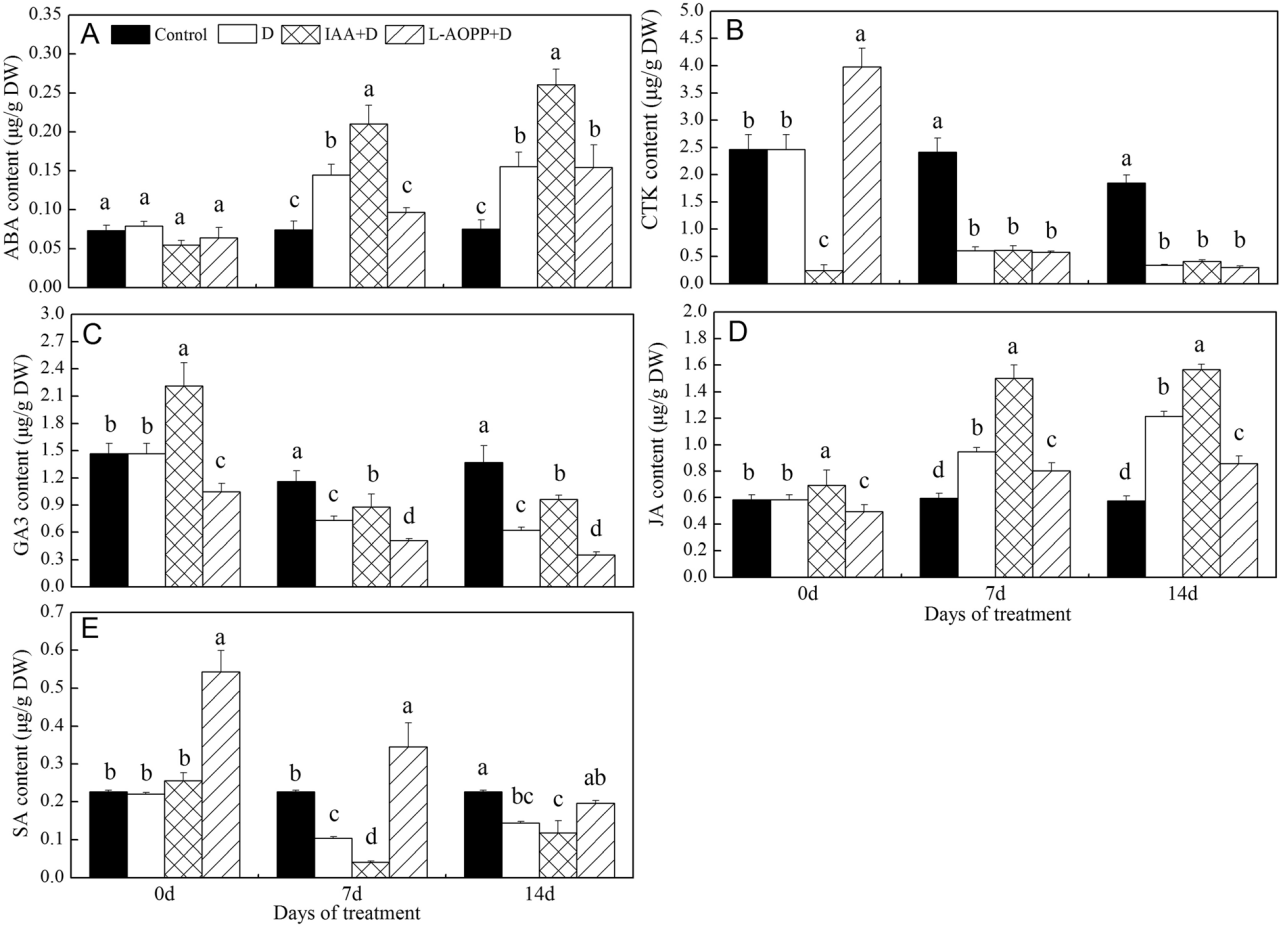
Content of other major phytohormones of white clover leaves in different sets. ABA content (**a**), CTK content (**b**), GA content (**c**), JA content (**d**) and SA content (**e**). Vertical columns represent Mean+/−std (*n* = 4). The same letter indicates no significant a difference and the different letters indicate significant difference (Fisher’s LSD, *P* < 0.05) in a pairwise comparison between sets at the same time

On day 0, the IAA reduced CTK by 90.2% and increased GA3 and JA by 45.2 and 18.4%, respectively (Fig. 2b-d). Nevertheless, it has no effect on ABA and SA content (Fig. 2a, e). However, L-AOPP increased CTK by 61.4% and SA by 130% (Fig. 2b, e), and reduced GA3 and JA contents by 28.8 and 13.8% (Fig. 2c, d). Meanwhile, it does not affect ABA content (Fig. 2a).

On days 7 and 14 (PEG stress), in all PEG treatments, all ABA, GA3, and JA in IAA pre-treatment were significantly higher than those in other treatment groups (Fig. 2a, c, d), while the SA content was significantly lower than that in other groups (Fig. 2e). In contrast, L-AOPP pre-treatment reduced the content of ABA, GA3 and JA (Fig. 2a, c, d) and increased the content of SA (Fig. 2e). Interestingly, CTK is not affected by IAA and L-AOPP.

### Endogenous IAA content and relative the expression of auxin-responsive genes

**Fig. 3:**
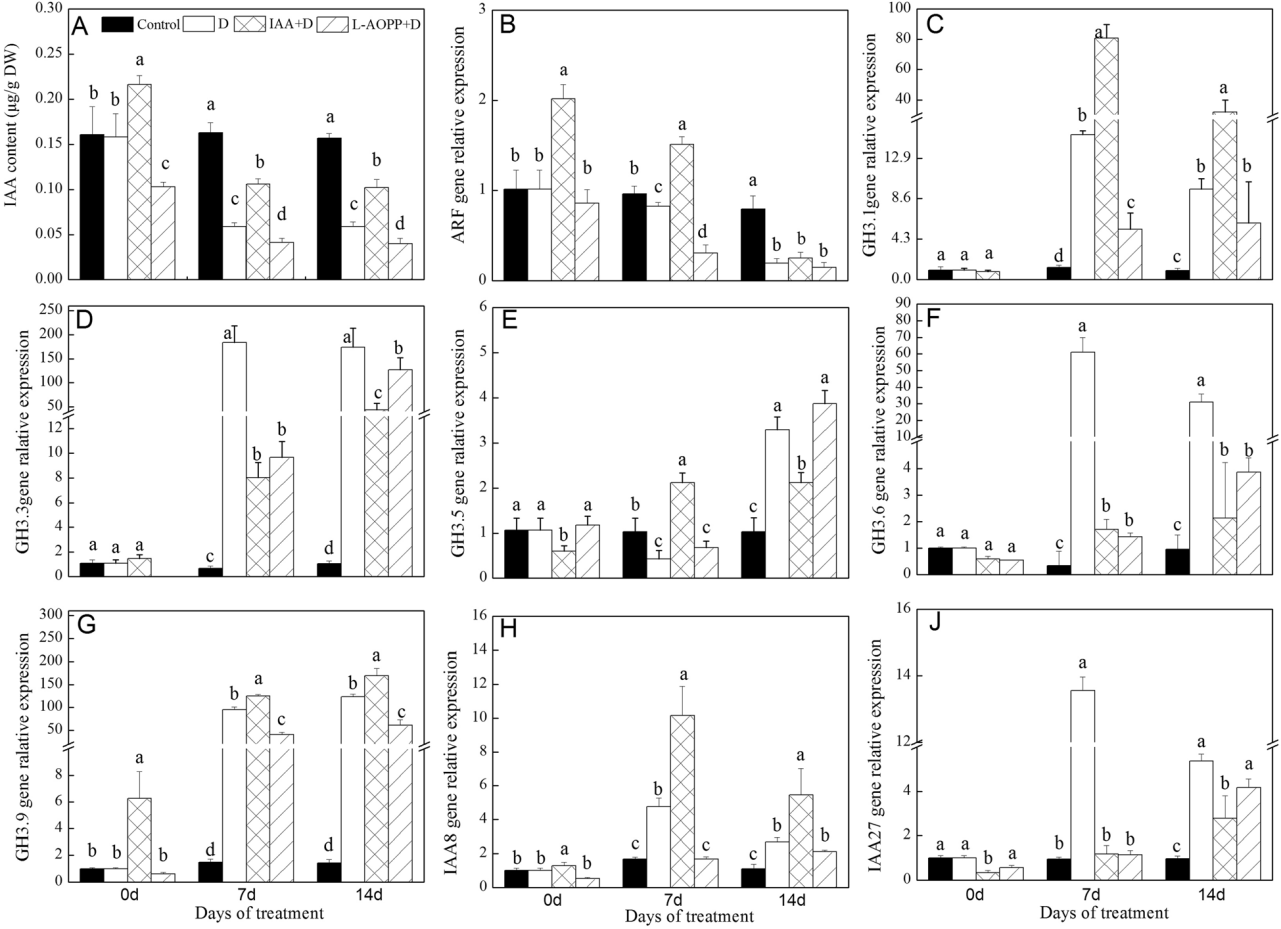
Endogenous IAA content (**a**) and relative expression of auxin response genes of white clover in different sets. *ARF* (**b**), *GH3.1* (**c**), *GH3.3* (**d**), *GH3.5* (**e**), *GH3.6* (**f**), *GH3.9* (**g**), *IAA8* (**h**), *IAA27* (**j**) and Vertical columns represent Mean + STD (*n* = 4). The same letter indicates no significant difference and the different letters indicate a significant difference (Fisher’s LSD, *P* < 0.05) in a pairwise comparison between sets at the same time

Clearly, the endogenous IAA content in control remained stable at all sampling times (Fig. 3a). On day 0, exogenous IAA increased the endogenous IAA content of white clover, while L-AOPP decreased it (Fig. 3a). On days 7 and 14, the content of endogenous IAA decreased significantly in groups treated with PEG (Fig. 3a), the content of endogenous IAA increased in the IAA pre-treatment group, while decreased in the L-AOPP group (Fig. 3a).

On day 0, exogenous IAA improved expression of *ARF*, *GH3.9* and *IAA8* (Fig. 3b, g, h), and decreased the expression of *GH3.5* and *IAA27* (Fig. 3e, j), but did not affect the expression of *GH3.1*, *GH3.3*, and *GH3.6* (Fig. 3c, d, f). L-AOPP inhibited the expression of *GH3.1* and *GH3.3* (Fig. 3c, d), but did not affect the expression of other genes.

On days 7 and 14, in all PEG treatments, IAA significantly increased the expression of *GH3.1*, *GH3.9* and *IAA8* (Fig. 3c, g, h), and decreased the expression of *GH3.3*, *GH3.6*, and *IAA27* (Fig. 3d, f, j). However, L-AOPP significantly down-regulated the expression of all genes except for *GH3.5* (Fig. 3b-j).

### The expression of transcription factor (TF) genes responding to drought

**Fig. 4:**
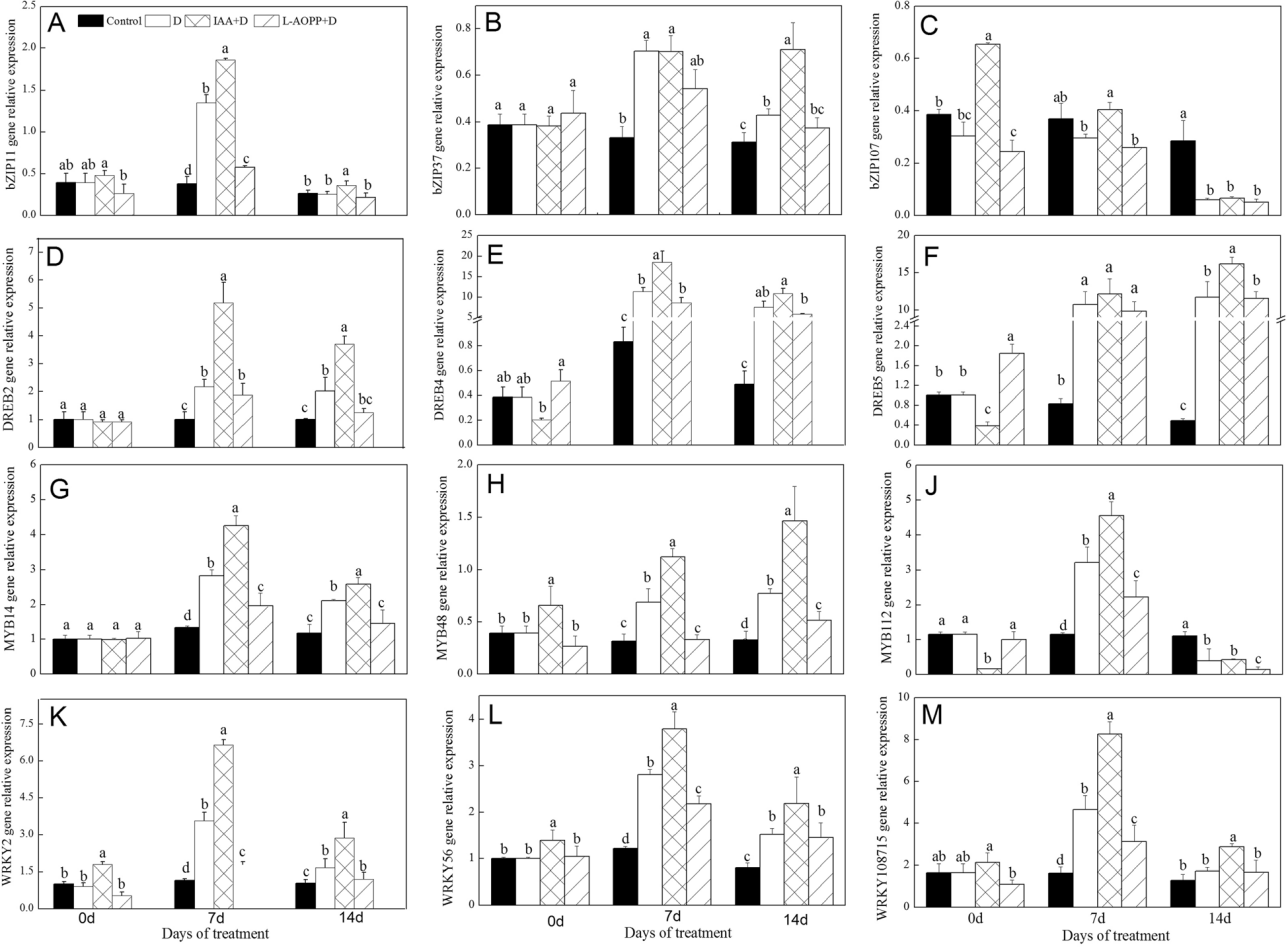
Expression of drought-induced transcriptional factors of white clover leaves in different treatments. *bZIP11* (**a**), *bZIP 37* (**b**), *bZIP 107* (**c**), *DREB2* (**d**), *DREB4* (**e**), *DREB5* (**f**), *MYB14* (**g**), *MYB48* (H), *MYB112* (**j**), *WRKY2* (**k**), *WRKY56* (**l**) and *WRKY108715* (**m**). Vertical columns represent Mean + STD (*n* = 4). The same letter indicates no significant difference and the different letters indicate a significant difference (Fisher’s LSD, *P* < 0.05) in a pairwise comparison between sets at the same time

In this experiment, we selected 3 genes from the *bZIP*, *DREB*, *MYB*, and *WRKY* transcription factor families, respectively. On day 0, IAA significantly up-regulated the expression of *bZIP107*, *MYB48*, *WRKY2* and *WRKY56* (Fig. 4c, h, k, l), and down-regulated the expression of *DREB5* and *MYB112* (Fig. 4f, j), but did not affect the expression of *bZIP11*, *bZIP37*, *DREB2*, *DREB4*, *MYB14* and *WRKY108715*. L-AOPP reduced the expression of *bZIP107* (Fig. 4c) and increased the expression of *DREB5* (Fig. 4f).

In all PEG treatments, on days 7 and 14, IAA up-regulated the expression of all transcription factor genes in most cases, while L-AOPP reduced the expression of these genes except *bZIP37* and *bZIP107* (Fig. 4b, c).

### Expression of drought-response genes and senescence-associated genes

**Fig. 5:**
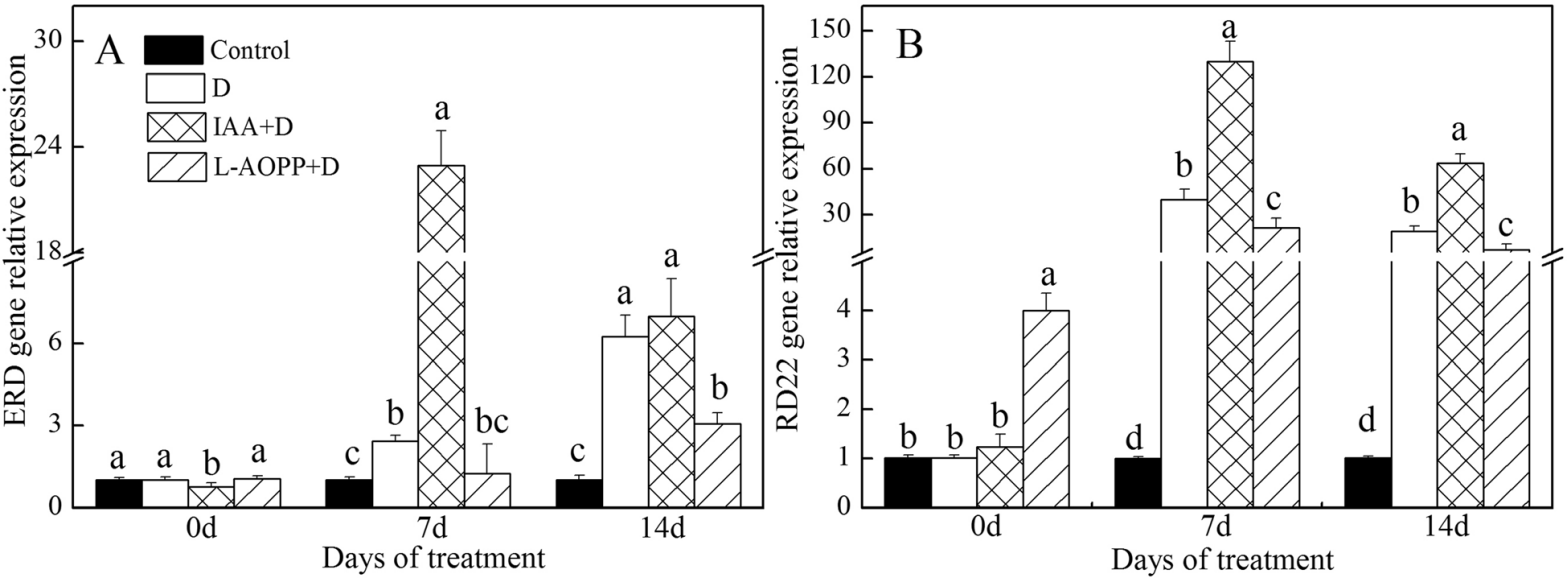
Expression of drought-induced genes of white clover leaves in different treatments. *ERD* (**a**) and *RD22* (**b**). Vertical columns represent Mean + STD (*n* = 4). The same letter indicates no significant difference and the different letters indicate a significant difference (Fisher’s LSD, *P* < 0.05) in a pairwise comparison between sets at the same time

**Fig. 6:**
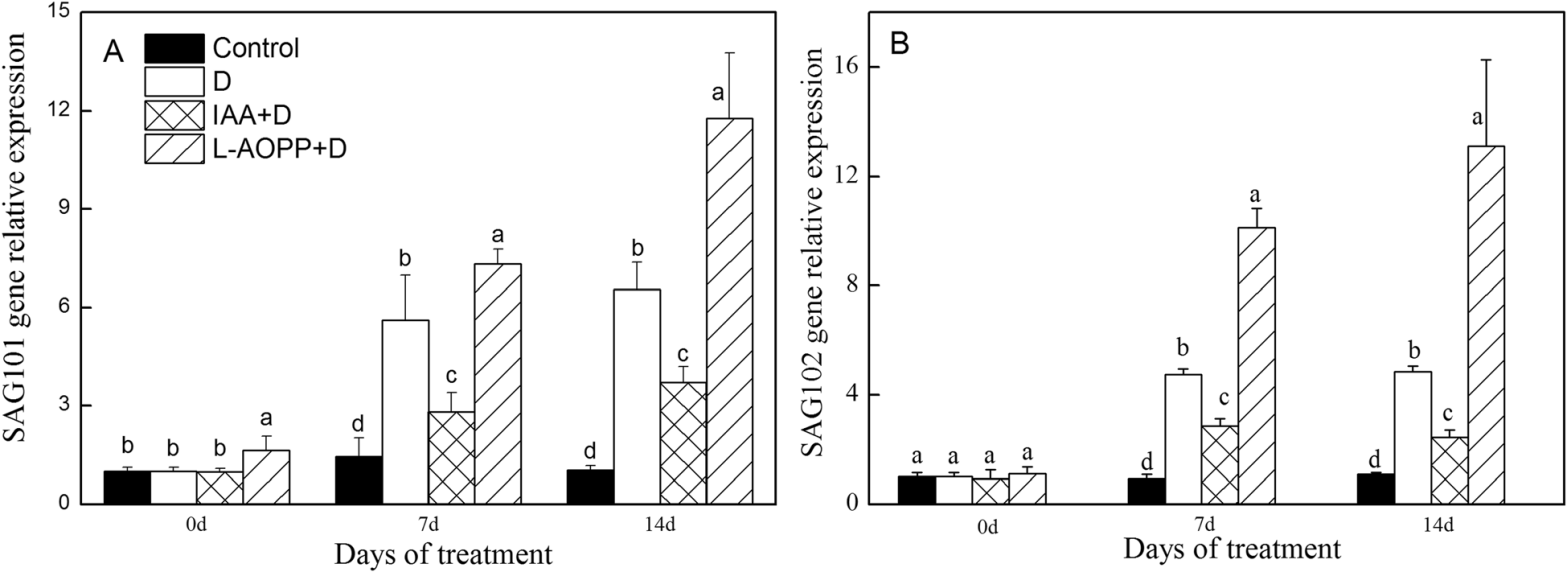
Expression of senescence-associated genes of white clover leaves in different treatments. *SAG101* (**a**) and *SAG102* (**b**). Vertical columns represent Mean + STD (*n* = 4). The same letter indicates no significant difference and the different letters indicate a significant difference (Fisher’s LSD, *P* < 0.05) in a pairwise comparison between sets at the same time

On day 0, exogenous IAA decreased the *ERD* expression (Fig. 5a), and L-AOPP improved the expression of *RD22* and *SAG101* (Figs. 5b, 6a). Both IAA and L-AOPP had no effects on the expression of SAG102.

On days 7 and 14, in all PEG treatments, IAA significantly enhanced the expression of *ERD* and *RD22* (Fig. 5a, b), and significantly decreased the expression of *SAG101* and *SAG102* (Fig. 6a, b). L-AOPP, however, significantly decreased the expression of *ERD* and *RD22* (Fig. 5a, b), and significantly increased the expression of *SAG101* and *SAG102.*

## Discussion

### Effects of IAA on white clover under no PEG stress

In the absence of PEG stress, IAA significantly increased stem dry weight of white clover, indicating that IAA pre-treatment caused white clover to accumulate more organic matter during the same growth time. At this time, the chlorophyll content of white clover leaves also increased significantly. Subsequently, increased content of chlorophyll contributed to an increase in photosynthetic rate.

As far as IAA and GA were concerned, some researchers proved that an average level of bioactive GA1 required normal level of IAA in elongating pea stems [36], and IAA promoted GA1 synthesis [37]. Here, our results showed that exogenous IAA increased GA3 (Fig. 2c). This probably because of IAA’s activation in enzymes related to GA3 syntheses, like IAA’s promotion on GA1 synthesis [37].

In zinnia, the transcript level of *IAA8* was induced by auxin [38]. Here, we found that exogenous IAA up-regulated the expression of *IAA8* in white clover. Therefore, it may be true that IAA is essential for the expression of *IAA8* gene. *IAA8* was found to play a crucial role in floral organ development and abnormal formation of floral in *IAA8*-mutation Arabidopsis could be changed through JA application, meaning that a strong link between *IAA8* and JA [39]. Fortunately, exogenous IAA significantly increased the expression of *IAA8* and JA levels in our findings. For the *ARF* gene, IAA significantly increased the expression of it in the present study. One study also found that auxin treatment could affect the transcript abundance of several *OsARF* genes, and these *ARF* genes might play crucial roles in varied metabolic pathways and some cellular processes in rice [40].

Several studies also showed that the high expression level of *ERD* and *RD22* subserved plant resistance to drought [41, 42]. *ERD* genes are a group of genes that are rapidly induced (in 1 h) under stress [43]. Our results showed that IAA significantly reduced the expression of *ERD* gene. It may be inferred that *the ERD* gene was not necessary for plants suffering no PEG stress, and IAA also inhibited the expression of it.

### Improved growth and physiologies in white clover under PEG stress

Under PEG stress, the results showed that exogenous IAA mitigated plant dehydration, and L-AOPP worsened it (Fig. 1a). IAA improved stem dry weight, relative water content, and total chlorophyll content in leaves, however, L-AOPP decreased all of them (Fig. 1b-d). Studies have shown that IAA is related to drought tolerance in plants, and wild Arabidopsis plants pre-treated with IAA exhibited enhanced drought resistance [44]. The application of IAA could ease the adverse effects brought by PEG stress and enhanced barley growth [45]. IAA conferred white clover with the better morphological and physiological state in the IAA + D group than that in the D group (Fig. 1), suggesting that IAA had a positive effect in improving drought tolerance of white clover.

### Changes in Phytohormones and subsequent effects in white clover under PEG stress

Transcriptome data revealed that an increase of ABA content activated expression of many drought-resistant genes [46]. ABA regulated downstream response of *RD29B* (dehydration stress gene) by regulating the *bZIP* gene [47]. In our studies, we also found that there was a consistent correlation between ABA and expression of *RD22* under PEG stress (Figs. 2a, 5b), suggesting that ABA also probably regulated expression of *RD22* gene in white clover and increased drought resistance.

It has been found that the interaction between IAA and ABA promoted the development of lateral roots in plants, and this pattern of root growth regulation was necessary for plants to respond to severe drought stress [48]. Besides, exogenous ABA enhanced the recovery of photosynthetic rate in upland rice under PEG stress [49]. Based on these experimental results and Figs. 1 and 2 A, we could speculate that an increase in ABA content might enhance drought resistance through multiple ways, such as improved *RD22* expression, higher total chlorophyll, and more stem dry weight. Moreover, the opposite effects L-AOPP on these indicators further confirmed that these changes arose from IAA.

GA3 application reduced cell permeability and electrolyte leakage under drought stress [50]. Therefore, increased content of GA3 could enhance drought resistance in plants. In the present study, the results showed that IAA significantly increased the content of GA3 in white clover under PEG stress, and improved its resistance to PEG stress.

And the strong interaction between JA and ABA was observed [51]. Some researchers have shown that JA was upstream of ABA biosynthesis, and the accumulation of JA at an early stage led to an accumulation of jasmonic acid isoleucine, which was one necessary condition for ABA synthesis under drought stress [52]. Here, our results showed that exogenous IAA increased the content of both JA (Fig. 2d) and ABA (Fig. 2a). And we could conclude that IAA might regulate ABA synthesis via JA in white clover.

In summary, we believed that plant hormones had reached a new homeostasis after applying exogenous IAA under PEG stress. Changes in these plant hormones may promote plant drought resistance through specific signal transduction and gene regulatory pathways.

### Expressions of genes responding to IAA and TF genes under PEG stress

Transcriptome data showed that rice *AUX/IAA* genes were induced by exogenous IAA and drought [53]. *AUX/IAA1* in Sorghum was also up-regulated by drought [54]. In this experiment, PEG stress significantly increased the expression of *IAA8* and *IAA27*. Interestingly, IAA significantly increased the expression of *IAA8* rather than *IAA27.* It was found that *IAA8* was involved in lateral root formation in Arabidopsis [55]. White clover under PEG stress would instinctively improve the expression of *IAA8* to form more lateral root to get more water, and IAA may enhance its expression. Some researchers revealed that the *Sl-IAA27* gene was down-regulated by auxin [56]. Our result of *IAA27* was in line with this finding. Tomato transgenic plants with under-expression of the *Sl-IAA27* gene showed multiple phenotypes interrelated to vegetative growth. Here, the down-regulation of *IAA27* may have multiple effects on growth and root development in white clover.

*GH3* family genes were also involved in plants responding to biotic and abiotic stress. Our studies showed that expression of *GH3.1*, *GH3.3*, *GH3.6*, and *GH3.9* were induced by drought stress (Fig. 3c, d, f, and g), denoting that these *GH* family genes could respond to drought stress. Besides, exogenous IAA also prompted expressions of *GH3.1* and *GH3.9* genes (Fig. 3c and g), showing that these two genes may have a relation to endogenous IAA content. *Arabidopsis thaliana* seedlings pre-treated with IAA showed an improved drought tolerance, and a variety of expressions of *GH* genes related to stress were regulated by exogenous IAA [57]. It was found that decreased endogenous IAA content in rice mutants accompanied by a deficiency in carotenoid and transgenic plants over-expressing *OsGH3.2* showed the sensitivity to drought [22]. Activation of *OsGH3.13* enhanced drought resistance in Rice [58]. Exogenous IAA activated responsive gene *GH3.9* and resulted in the strong drought resistance in plants [59]. These results showed that exogenous IAA could enhance drought resistance in white clover, and *GH3.1* and *GH3.9* gene was involved in drought tolerance.

For *bZIPs*, only a few members were identified to play roles in plant growth and development, abiotic stress, and hormone signal transduction, but their potential molecular mechanisms were still unknown and need further exploration [60]. An earlier study has shown that *OsbZIP23* in maize is involved in ABA signaling and regulates drought stress [61]. Other researchers found that *bZIP11* in Arabidopsis interacted with one adapted proteins via an amino-terminal activation domain to recruit the histone acetylation system to specific auxin-responsive genes [62]. *bZIP37* expressed in the salt-stressed plant activating downstream of ABA-induced gene expression [63]. We also found that expression of *bZIP11* was also induced by exogenous IAA (Fig. 4a), and that of *bZIP37* was induced by PEG-6000 (Fig. 4b).

Also, *DREBs* (dehydration-responsive element-binding proteins) play essential roles in plant response to drought stress and were found to be activated in ways dependent on ABA [64]. It was shown that exogenous IAA enhanced expression of *DREB2* and *DREB4*, and L-AOPP negatively regulated expression of *DREB2* and *DREB4* (Fig. 4d, e) in our studies. Another study has shown that *DREBs* regulating the expression of many downstream genes of drought resistance and over-expression of the *DREB* gene can enhance drought resistance in plants [65]. Our results showed that improved drought resistance of white clover by exogenous IAA could be associated with the expression of the *DREB2* and *DREB4*.

*MYBs* are also essential in regulating plant growth, development, metabolism, and stress response, and almost all eukaryotes have *MYB* transcription factors. The response mechanisms of *MYBs* in the stress environment are not very clear. Our studies found that both exogenous IAA and drought stress-regulated expression of *MYB14* and *MYB48* and L-AOPP decreased their expressions (Fig. 4g, h). Xiong found that the over-expression of *MYB48–1* promoted biosynthesis of ABA and improved drought resistance of transgenic rice [29]. *AtMYB60* regulated stomatal movement and promoted *Arabidopsis thaliana* to respond to drought stress [66]. Different *MYBs* showed varied functions in the progress of responding to drought and improved drought resistance.

At present, research of *WRKY* transcription factors in abiotic stress has been progressed. It was found that *WRKYs* were involved in plant stress regulatory networks, and *WRKY* proteins were induced by drought stress [67]. *WRKYs* also played essential roles in plant drought stress and regulated plant response to abiotic stress through interaction with hormones and protein kinases [68]; however, the molecular mechanisms of their regulations were still limited. *WRKY* transcription factor *ABO3* induced expression of drought resistance genes, such as *RD29A* and *COR47*, and improved drought resistance [69]. In terms of *WRKY2*, *WRKY56*, and *WRKY108715* gene, we found that drought induced their expressions, and exogenous IAA also up-regulated the expression of *WRKY* family genes in white clover.

### Expression of stress gene and senescence-associated gene under PEG stress

*ERD* and *RD22* subserved plant resistance to drought [41, 42], and our results suggested that both PEG stress and exogenous IAA up-regulated the expression of them in white clover, but L-AOPP decreased them. Besides, the expression of *ERD11* and *ERD13* gene was induced by dehydration, but not influenced by GA, ABA, 6-BA, and 2,4-D [70]. Other studies also found that the *RD22* gene was doubled by both ABA and MYB proteins [71].

As far as *SAG101* and *SAG102* gene was concerned, our study found that PEG stress and L-AOPP increased their expressions, but IAA decreased them. *SAG101* in Arabidopsis encoded an Acyl hydrolase involved in leaf senescence [72]. It was found that exogenous IAA inhibited the transcription level of *SAG12* [73] and retarded the senescence of leaves. The plant with over-expression of the *YUCCA6* gene improved the content of endogenous IAA and hindered senescence of plant by down-regulating expression of *SAG12* [74]. Similarly, the decreased expression of *SAG101* and *SAG102* by IAA could play a role in delaying senescence resulted from PEG stress in white clover.

## Conclusion

Above all, this study highlighted the protective role of exogenous IAA during drought tolerance in white clover. Drought responsive plant hormones, such as ABA, JA and GA, transcription factors (*bZIP11*, *DREB2*, *MYB14*, *MYB48* and *WRKYs*,) and related genes (*ERD*, *RD22*, *SAG101* and *SAG102*) could play a positive role in alleviating drought stress damage .

## Methods

### Plant material and growth condition

Seeds of ‘Pixie’ (*Trifoliumrepens cv*.) were purchased from Beijing Mammoth Seed Industry Company, Beijing, China. The seeds were sterilized with 1% (w/v) sodium hypochlorite solution for 3 min and then rinsed 3 times with sterile water. The seeds were planted in pots (9 cm depth, 18 cm width, 24 cm length) filled with sterilized quartz sand. The pots were placed in a growth chamber (23 °C/16 h during the day and 19 °C/8 h at night, with an irradiance of approximately 300 μmol quanta·m^− 2^·s^− 1^, and relative humidity of 75%) and 50 mL deionized water was added daily in each pot. After germination, seedlings were transplanted in Hoagland’s nutrient solution. Positions of the pots were rearranged daily in order to reduce the environmental impact.

### Experimental design

The seedlings at the two-leaf stage were divided into four groups: (1) control, Hoagland’s nutrient solution; (2) PEG-6000 treatment (labeled as D), Hoagland’s nutrient solution containing 15% PEG-6000 (W/V,-0.3Mpa); (3) IAA pre-treatment + PEG-6000 treatment (labeled as IAA + D), first pre-treated with Hoagland’s nutrient solution containing 1 μM IAA for 7 days and then added 15% PEG-6000 in Hoagland’s nutrient solution; (4) L-AOPP pre-treatment+PEG-6000 treatment (labeled as L-AOPP+D), first pre-treated with Hoagland nutrient solution containing 100 μML-AOPP for 7 days and then added 15% PEG-6000 in Hoagland’s nutrient solution. Each treatment had four biological replications. Samples were taken at 0 (labeled with 0 d), 7 (labeled as 7 d) and 14 (labeled as 14 d) days after treating with PEG-6000.

### Measurement of physiological indicators

#### Relative water content (RWC), stem dry weight and Total chlorophyll content (Chl)

For RWC, the 0.2 g fresh leaves were taken as fresh weight (FW). Then leaves were placed in distilled water at 4 °C for 24 h and weighed to get saturated weight (SW). Subsequently, samples were dried at 105 °C for 30 min, followed by drying at 75 °C for 48 h and weighed to record dry weight (DW). RWC was calculated by the formula: RWC(%) = (FW-DW)/ (SW-DW) × 100% [75].

The total chlorophyll contents were extracted by incubating ~ 0.1 g fresh leaves with a 10 mL solution of 80% acetone: 95% methanol (1:1, V/V) in the dark until the leaves became colorless. The light absorption values of Chl a and Chl b were measured at 645 nm and 663 nm, respectively. The total chlorophyll contents were calculated according to the formula:

Chl (a + b)(mg/g) = (20.2 × OD_645_ + 8.02 × OD_663_)/(DW × 1000) [75].

#### Quantification of IAA, ABA, CTK (iPAs and ZRs), GA3, JA and SA

HPLC–ESI–MS method (minor modification) [76] was used to quantify the phytohormones. 0.2 g leaves were transferred to a 5 ml screw-cap tube, freeze in liquid nitrogen for 10 min and grounded to powder form using Plant Tissue Breaker. Firstly, 200 μl working solution of internal standards was added to each tube, then 2 mL extraction solvent was added and shook at the rate of 100 rpm for 30 min at 4 °C; subsequently, 2 mL dichloromethane was added to each tube and shook as before. Then samples were centrifuged at the rate of 12,000 rpm for 5 min at 4 °C and transferred ~ 1.8 ml of the solvent from the lower phase into a screw-cap vial followed by concentrating through nitrogen evaporator. For analysis, samples were redissolved with 0.2 mL methanol by injecting 20 μl of the methanol solution into the C18 (reverse-phase) HPLC column. The HPLC–ESI–MS (HPLC system, LC-10 AD series, Shimadzu, Japan; ESI-MS system, ABI 4000 QTRAP) conditions and settings were employed as reported previously [76]. Applied Biosystems Analyst software version 1.5.1 was used to control the MS system and to perform data analysis and data management.

### Expression analysis of genes by realtime qRT-PCR

Real-time quantitative PCR (qRT-PCR) was used to test the expression of *GH3.1*, *GH3.3*, *GH3.5*, *GH3.6*, *GH3.9*, *IAA8*, *IAA27*, and *ARF* in leaf. Total RNAs of the leaf was extracted through Plant RNA Kits (TIANGEN Biochemical Technology Co., Ltd), and cDNAs used in qRT-PCR were synthesized using iScriptTM cDNA Synthesis Kit (Bio-Rad Laboratories, Inc). 10 μL qRT-PCR reaction system consisted of 1 μL cDNA, 0.5 μL each of upstream and downstream primers, 5 μL SYBR Green SuperMix and 3 μL ddH2O. qRT-PCR program was: pre-denaturation 30 s at 95 °C, denaturation 10 s at 95 °C, annealing 15 s at 59.3 °C (*GH3.1*), 61 °C (*GH3.5*), 60 °C (*GH3.3*, *GH3.6*, *GH3.9, IAA8*, *IAA27*) and 58.9 °C (*ARF*), extension 5 s at 72 °C, 40 cycles, then last extension 10 min at 72 °C. *GAPDH* was used to calculate the relative expression level of each concerned gene through the formula of 2^-ΔΔCt^ [77]. Primer sequences of the genes related to IAA and their corresponding GeneBank accession numbers were listed in Additional file 1: Table 1.

10 μL qRT-PCR reaction system was the same as mentioned above. The reaction program was: pre-denaturation 30 s at 95 °C, denaturation 10 s at 95 °C, annealing 15 s at 57.2 °C (*bZIP37、bZIP107、MYB48、MYB112*), 58 °C (*DREB2、DREB4、DREB5 、MYB14)*, 58.4 °C (*WRKY108715*), 61 °C (*WRKY2*、*WRKY56、bZIP11)*, 56.4 °C (*RD22)* and 59.5 °C (*ERD*), 55.5 °C (*SAG101/SAG102*), extension 5 s at 72 °C, 40 cycles, then last extension 10 min at 72 °C. The reference gene also was *GAPDH*, and calculation was the formula of 2^-ΔΔCt^ [77]. Primer sequences of drought-induced transcription factors and drought-induced genes and their corresponding Gene Bank accession numbers were listed in Additional file 1: Table 2.

### Statistic

In this paper, Origin 8.5.1 was employed to generate the histograms, SPSS 19.0 to the analysis of variance (ANOVA) at the 0.05 probability level. Data were transformed to meet normality and homogeneity of variance. Fisher’s LSD was used to determine differences between groups.

## Supplementary information

**Additional file 1: Table 1** Primer sequences of the genes related to IAA and their corresponding GeneBank accession numbers. **Table 2** Primer sequences of drought-induced transcription factors and drought-induced genes and their corresponding GeneBank accession numbers.

## Data Availability

The data used and/or analyzed during the current study are available from the corresponding author on reasonable request.

## Abbreviations

IAA: Indole-3-acetic acid
L-AOPP: L-2-aminooxy-3-phenyl propionic acid
ABA: Abscisic acid
JA: Jasmonic acid
CTK: Cytokinin
GA(3): Gibberellin(3)
SA: Salicylic acid
GAPDH: Glyceraldehyde-3-phosphate dehydrogenase
FW: Fresh weight
SW: Saturated weight
DW: Dry weight
ERD: Early-response to dehydration
RD: Response to dehydration
SAG: Senescence-associated genes
TF(s): Transcriptional factor(s)
bZIP: Basic region/leucine-zipper
DREB: Dehydration responsive element binding
MYB: Myeloblastosis
ARF: Auxin response transcription factor
PEG-6000: Polyethylene glycol-6000
RWC: Relative water content
Chl: Total chlorophyll content
