# Supplementary material for: Indole-3-acetic acid improves drought tolerance of white clover via activating auxin, abscisic acid and jasmonic acid related genes and inhibiting senescence genes
Source: BMC Plant Biol. 2020 Apr 8;20:150. doi: 10.1186/s12870-020-02354-y (PMC7140375; doi:10.1186/s12870-020-02354-y)
Supplement: Supplementary file 1 — Additional file 1: Table 1 Primer sequences of the genes related to IAA and their corresponding GeneBank accession numbers. Table 2 Primer sequences of drought-induced transcription factors and drought-induced genes and their corresponding GeneBank accession numbers. [file 12870_2020_2354_MOESM1_ESM.doc]

**Additional file**

**Tables**

Table 1 Primer sequences of the genes related to IAA and their corresponding GeneBank accession numbers

| Target gene | Accession No. | Forward primer (5'-3') | Reverse primer (5'-3') |
| --- | --- | --- | --- |
| GH3.1 | MF099746 | TCGTCAACTTCTATACAGCCTTCT | CACTTGGTGTCCTTGTTTCTGA |
| GH3.3 | MF099747 | TGACTCGGACAAAACAGACG | CTTCATCACTAGGTGGATTAGAAG |
| GH3.5 | MF099748 | GATGCTGAGAATGTTCAAAAGG | AGAAACATCACCACCAACCA |
| GH3.6 | MF099749 | GAAGAAGAGTTAGGGAGGAGAAG | CCAGGTGTTTTAGCCTCAGAT |
| GH3.9 | MF099750 | CATTGAAGCAGTGGTTACAGG | CACCAAAGTAACACTCAGAAGAAG |
| IAA8 | MF099751 | ATGCTATCGCCTAGACCTGTT | TGCCTTAGATGCTGGCTGTG |
| IAA27 | MF099752 | CCTCAAAGCTACTGAACTGAGAC | ACCCATTTACCAGAACCTCC |
| ARF | MF099753 | TCTGCTGAGTTTACGAGGGTTC | GGTTTTGTTGCTTGCTGATGC |
| *GAPDH* | F968420.1 | TTACAGAAAGGCACAGGGATGAC | CGGGAGACTAAGGAGGAACTAT |

Table 2 Primer sequences of drought-induced transcription factors and drought-induced genes and their corresponding GeneBank accession numbers

| Target gene | Accession No. | Forward primer (5'-3') | Reverse primer (5'-3') |
| --- | --- | --- | --- |
| DREB2 | EU846194.1 | CAAGAACAAGATGATGATGGTGAAC | AAGAAGAAGAATTGGAGGAGTCATG |
| DREB3 | EU846196.1 | GCTCAATAGGACTCAACCAACTCAC | TGACGTTGTCTAACTCCACGGTAA |
| DREB4 | EU846198.1 | CTTGGTTGTGGAGATAATGGAGC | AAGTTGCAATCTGAATTCTGAGGAC |
| DREB5 | EU846200.1 | GCGATAGGTTCAAAGAAAGGGTG | AGAGCAGCATCTTGAGCAGTAGG |
| bZIP11 | MF099755 | TTCCTTGCCTCCACTTAGTCC | GATCGTCTGTGCCCTTTACG |
| bZIP 37 | MF099754 | GAACCCGTCTGAACATAACTGAA | AGCGACTTTGGAGCCATCAT |
| bZIP 107 | MF099756 | AGACCCACCAATAACCAAACTG | CATAAAAGGAAGAAGAAGGAGGAG |
| MYB14 | JN117923.1 | GACGAAGAGAAAGAACTATCCGCA | TTGATCCGAACAAGGCGACA |
| MYB48 | MF099757 | CGAGAAAGGTCATACAAACAAAGG | TGAGGTCAGGGCGGAGATAG |
| MYB112 | MF099758 | GCCAGGAAGAACCGACAATG | GCCAGGAAGAACCGACAATG |
| WRKY2 | MF099759 | GGCACATAACCACCCGAAAC | AAATTAGCCCAGCCACGATC |
| WRKY56 | MF099760 | GCTCTTTTGCTCCAAGCTGTC | AATTGAGGCTCACGCTACGG |
| WRKY108715 | MF099761 | GAACAGACCAACTCCAAACAGC | GCAAATCAGGATGGAAAGGAC |
| ERF019 | MF099762 | GATATTGCTATGGATGTCGATGC | AAGTCCTCTTGTTGGCTAGAAACT |
| ERF098 | MF099763 | TGCGGCGGAGATACGAGAT | GGAAGAAGTGGGCTTAGAAGGA |
| ERF110 | MF099764 | TTCGCCATCGCTTTCTTTGT | TCCGCTACGAGATTGATCTTCC |
| ERD | XM_003612152.2 | CCATCGCTGTCTATGCTCGTA | TTCTTCCTCGTCTGAATCGGTA |
| RD22 | XM_003588503.2 | GTCCAAACTTCCCACAACTCA | CCTCCTTTTCCTACAGCTACTG |
| GAPDH | JF968420.1 | TTACAGAAAGGCACAGGGATGAC | CGGGAGACTAAGGAGGAACTAT |
| SAG101 | XM_004489275.2 | CATTTCGTACTCGCTGGCTCT | CACGTAATCCTTACCACCGTCT |
| SAG102 | XM_003590568.2 | ATCATTGGACTTGGTCTTGTTGG | GAAGTGGGCAAGGGAGGAAT |
